# Management of a Late-Term Hiatal Hernia with Intrathoracic Pouch Migration After Roux-en-Y Gastric Bypass

**DOI:** 10.1007/s11695-021-05881-1

**Published:** 2022-01-06

**Authors:** Theodoros Thomopoulos, Maurice FitzGerald, Styliani Mantziari, Nicolas Demartines, Michel Suter

**Affiliations:** 1 Department of Visceral Surgery, University Hospital of Lausanne (CHUV), Rue du Bugnon 46, 1011 Lausanne, Vaud Switzerland; 2 Faculty of Biology and Medicine, University of Lausanne, Lausanne, Switzerland; 3 Department of Surgery, Riviera-Chablais Hospital, Rennaz, Switzerland

**Keywords:** Hiatal hernia, Roux-en-y gastric bypass, Fundopli cation, Laparoscopic revision

## Introduction

Hiatal hernia (HH) with intrathoracic migration of the gastric pouch (IMGP) is a rare long-term complication of laparoscopic Roux-en-Y gastric bypass (LRYGB); however, its management remains poorly documented [1, 2]. Several factors may contribute to the development of this entity. Rapid weight loss can lead to the relaxation of the phreno-esophageal ligament and enlargement of the hiatus [3] allowing for easier migration. Creation of a large gastric pouch could favor its migration because of the higher intra-gastric pressure [4]. Extended dissection of the cardia and the left crus during the LRYGB may disrupt normal attachments between the cardia and diaphragm and predispose to IMGP [5]. Finally, a small sliding HH may pass unperceived pre- or even intra-operatively, or intentionally not repaired during LRYGB, leading to progression of HH after bariatric surgery [6].

Patients with IMGP may present with epigastric/retrosternal pain, dysphagia, and regurgitations. In addition to the conventional imaging techniques used for the diagnosis of IMGP, such as CT scan and barium transit, the swallow magnetic resonance imaging (MRI) has been recently described as a new dynamic method to assess the anatomy of the gastric pouch, as well esophageal dysmotility after LRYGB, and could be of great use in the diagnostic armamentarium of swallowing difficulties after RYGB [7]. Various surgical techniques have been described in the literature to address HH and IMGP after LRYGB, yet these reports are sporadic and inconsistent. Among them, the hiatoplasty with the ligamentum teres hepatis [8], or a modified Nissen procedure using the fundus of the excluded stomach, may be valid options [9].

The purpose of the present work is to illustrate the main technical aspects of a laparoscopic-modified Nissen fundoplication with the excluded stomach to correct IMGP after RYBG.

## Methods

This video shows the case of a 54-year-old female patient, with an ante-colic LRYGB performed 14 years earlier in our institution for an initial BMI of 46 kg/m^2^. The patient lost 45 kg during the first 2 years after surgery but regained almost 20 kg over the last 5 years with a current BMI of 38 kg/m^2^. She presented with epigastric pain, reflux, and dysphagia to solid food with occasional postprandial vomiting. Although upper endoscopy did not reveal any anatomical anomaly, a barium swallow and a thoraco-abdominal CT scan showed herniation of the gastric pouch with an associated kinking effect at the level of the hiatus. Surgical management of the IMGP was therefore proposed.

## Results

The laparoscopic exploration, lysis of adhesions, reduction of the HH, posterior crural repair, and the modified Nissen fundoplication with the gastric remnant are demonstrated step-by-step in this video. The postoperative course was uneventful and she was discharged on POD3. At 6-month follow-up, she no longer had any abdominal pain and gastric reflux, and food tolerance was excellent.

## Conclusion

IMGP is a well-known but rare complication of LRYGB, and bariatric surgeons need to be familiar with its management. In case of symptoms suggesting IMGP, a thorough pre-operative work-up is essential before proceeding to surgery. Surgical correction of IMGP with a modified Nissen fundoplication using the excluded stomach is a technically demanding procedure, but in experienced hands, it can be safely performed with minimal morbidity.

## Supplementary Information

Below is the link to the electronic supplementary material.

Supplementary file1 (MP4 116884 KB)
